# Human Engineered Cartilage and Decellularized Matrix as an Alternative to Animal Osteoarthritis Model

**DOI:** 10.3390/polym10070738

**Published:** 2018-07-04

**Authors:** Marta Galuzzi, Sara Perteghella, Barbara Antonioli, Marta Cecilia Tosca, Elia Bari, Giuseppe Tripodo, Milena Sorrenti, Laura Catenacci, Luca Mastracci, Federica Grillo, Mario Marazzi, Maria Luisa Torre

**Affiliations:** 1 Tissue Therapy Unit, ASST Niguarda Hospital, Piazza Ospedale Maggiore 3, 20162 Milan, Italy; marta.galuzzi@ospedaleniguarda.it (M.G.); barbara.antonioli@ospedaleniguarda.it (B.A.); martacecilia.tosca@ospedaleniguarda.it (M.C.T.); mario.marazzi@ospedaleniguarda.it (M.M.); 2 Department of Drug Sciences, University of Pavia, Viale Taramelli 12, 27100 Pavia, Italy; elia.bari@unipv.it (E.B.); giuseppe.tripodo@unipv.it (G.T.); milena.sorrenti@unipv.it (M.S.); laura.catenacci@unipv.it (L.C.); marialuisa.torre@unipv.it (M.L.T.); 3 PharmaExceed S.r.l., 27100 Pavia, Italy; 4 Section of Histopathology, Department of Surgical Sciences and Integrated Diagnostics (DISC), IRCCS San Martino IST Hospital, University of Genoa, Largo R. Benzi 8, 16121 Genoa, Italy; luca.mastracci@unige.it (L.M.); federica.grillo@unige.it (F.G.)

**Keywords:** human chondrocytes, osteoarthritis, alginate, silk fibroin, beads, microcarrier, pellet, decellularized cartilage matrix

## Abstract

(1) Objective: to obtain a reproducible, robust, well-defined, and cost-affordable in vitro model of human cartilage degeneration, suitable for drug screening; (2) Methods: we proposed 3D models of engineered cartilage, considering two human chondrocyte sources (articular/nasal) and five culture methods (pellet, alginate beads, silk/alginate microcarriers, and decellularized cartilage). Engineered cartilages were treated with pro-inflammatory cytokine IL-1β to promote cartilage degradation; (3) Results: articular chondrocytes have been rejected since they exhibit low cellular doubling with respect to nasal cells, with longer culture time for cell expansion; furthermore, pellet and alginate bead cultures lead to insufficient cartilage matrix production. Decellularized cartilage resulted as good support for degeneration model, but long culture time and high cell amount are required to obtain the adequate scaffold colonization. Here, we proposed, for the first time, the combined use of decellularized cartilage, as aggrecanase substrate, with pellet, alginate beads, or silk/alginate microcarriers, as polymeric scaffolds for chondrocyte cultures. This approach enables the development of suitable models of cartilaginous pathology. The results obtained after cryopreservation also demonstrated that beads and microcarriers are able to preserve chondrocyte functionality and metabolic activity; (4) Conclusions: alginate and silk/alginate-based scaffolds can be easily produced and cryopreserved to obtain a cost-affordable and ready-to-use polymer-based product for the subsequent screening of anti-inflammatory drugs for cartilage diseases.

## 1. Introduction

Osteoarthritis is a highly prevalent degenerative osteoarticular disease that causes serious debilitating condition and the interest of the scientific community is oriented to the development of new cost-effective therapies [1]. The studies on animal models significantly contribute to understanding the mechanisms of this disease, but their actual effectiveness remains controversial, in particular in predicting the action of new drugs in humans. In fact, the differences between animals and humans limit the usability of rodents for the evaluation of efficacy, safety and toxicity of new therapeutics. Furthermore, the Directive 2010/63/EU of the European Parliament limits the use of animals for scientific purposes, as a result of the 3Rs principle, promoted by Russell and Burch in 1959 [2]. Animals must be replaced by alternative methods (Replacement), their number must be reduced to a minimum (Reduction) and, where not possible, procedures should minimize the suffering of the same (Refinement). In this contest, 3D cell cultures have been proposed to obtain predictive in vitro models of numerous pathologies; in particular, during the screening of new drugs, 3D models can be used to test a large number of bioactive compounds reducing the cost of the early stages of drug discovery [3,4,5]. 3D models can not totally substitute animal testing, but could be considered as a valid support to obtain more information about the efficacy of bioactive compounds [5].

In vitro models of osteoarthritis can be realized using engineered cartilage, based on cells and scaffolds. Chondrocytes are the only cells residing in cartilage, able to maintain the turnover of extracellular matrix through the secretion of glycoprotein, collagens, proteoglycans, and hyaluronan. Unfortunately, this cell type shows low proliferative ability, and loses its phenotype when cultured in monolayer conditions [6]. Therefore, three-dimensional models, able to mimic the physiological microenvironment, represent the only one promising approach to study the physiopathological mechanisms, as well as for new drug development [7]. In fact, scaffolds inhibit chondrocyte dedifferentiation, withstand the mechanical forces in vivo and allow the diffusion of nutrients, gases, and waste [8,9,10].

The aim of this work was to develop a reproducible, robust, well-defined, and cost-affordable tissue scaffold to produce a suitable model of cartilaginous pathology. In detail, osteoarthritis is an inflammatory condition that leads to a progressive degradation of cartilage matrix, due to aggrecanase expression. These enzymes cut aggrecan, the main proteoglycan that composes cartilage, with the release of glycosaminoglycan (GAG). Human articular and nasal chondrocytes were considered and different 3D models were tested: pellet culture of chondrocytes, decellularized cartilage matrix, and two alginate-based scaffold (beads and microcarriers). These scaffolds may replace the native matrix and allow the reduction of cells needed for single test. Engineered cartilages were subjected to the treatment with interleukin-1β, to define the best degeneration model. Finally, alginate-based 3D models were cryopreserved to store them and to obtain a ready-to-use device.

## 2. Materials and Methods

### 2.1. Materials

All reagents used for cell cultures were purchased from Euroclone (Milan, Italy). Collagenase, sodium alginate, sodium dodecyl sulfate, 3-(4,5-dimethylthiazol-2-yl)-2,5-diphenyltetrazolium bromide, 1,9-dimethylmethylene blue, DMSO, papain, interleukin-1β, sodium, and calcium chloride were obtained from Sigma-Aldrich (Milan, Italy).

### 2.2. Isolation and Culture of Human Articular and Nasal Septal Chondrocytes

For isolation of primary nasal septal chondrocytes (NCs), human nasal septal healthy cartilage was harvested during septoplasties or septorhinoplasties in Department of Otorhinolaryngology (I.R.C.C.S. Policlinico San Matteo, University of Pavia, Pavia, Italy). For NCs, we considered 20 informed donors (age 37.11 ± 15.81 years). Articular chondrocytes (ACs) were isolated from pathologic human knee cartilage during joint replacement and/or cleaning surgery after sport trauma (ASST Grande Ospedale Metropolitano Niguarda, Milan, Italy). Overall, for ACs we harvested tissues from eight informed donors (age 43.38 ± 14.31 years). Clinical sheets of donors (identity, gender, age, tissue processing, sampling site, day of collection and anamnesis) are filed by the surgery-responsible structure. Donors with septicemia or extensive infections, syphilis, hepatitis B and C, HIV, Creutzfeld-Jacobs disease, viral or unknown neurological diseases, human GH treatment, and malignant tumors were excluded from the trial. All subjects gave their informed consent for inclusion before they participated in the study. The study was conducted in accordance with the Declaration of Helsinki, and the protocol was approved by the Ethics Committee of ASST Grande Ospedale Metropolitano Niguarda (Milan, Italy) (Ref. 12.11.2009).

Each sample was suspended in phosphate buffered saline (PBS), weighed and digested with trypsin-EDTA 1X, for 30 min (37 °C, 5% CO_2_), followed by overnight incubation with 200IU type IA collagenase. Obtained suspension was filtered through 70 µm nylon mesh (Greiner Bio-One GmbH, Kremsmünster, Austria) and cells were centrifuged (300× *g*, 5 min). Chondrocytes were counted to determine cellularity (number of cell/grams of tissue) and seeded onto flasks (7000 cells/cm^2^) with Dulbecco’s Modified Eagle’s Medium High Glucose (DMEM-HG), 10% fetal bovine serum (FBS), penicillin (100 IU/mL), streptomycin (100 µg/mL), amphotericin B (2.5 µg/mL), fibroblast growth factor-2 (FGF-2, 10 µg/mL) and Transforming Growth Factor β1 (TGF-β1, 1 µg/mL). Expanded chondrocytes were used for experiments using a chondrogenic medium (supplemented of TGF-β1 10 µg/mL). Each cell line was used until third passage of culture.

To evaluate cellular doubling, replication rate and population doubling time, ACs and NCs were cultured (10,000 cells/cm^2^) from P0 to P3. For each cell source, six donors have been considered. Every seven days cells were detached and counted to calculate cellular doubling (CD) using the following equation: CD = log(*N*/*N*0) × log2, where *N* = number of counted cells and *N*0 = number of seeded cells. Replication rate (RR) and population doubling (PD) were also calculated using the equation: RR = *T*/log(*N*/*N*0) and PD = *T*/(log(*N*/*N*0) × log2), where *T* = culture time necessary to reach the sub-confluence, *N* = number of counted cells and *N*0 = number of seeded cells. Results were reported, for both ACs and NCs, as mean value ± standard deviation.

### 2.3. Preparation of Decellularized Cartilage

Samples of human septal nasal healthy cartilage were processed using a 2 mm dermal biopsy punch (B Life, Lughignano, Italy). Decellularization was made as reported by Kheir and colleagues [11], with some modifications. Briefly, two freeze-thaw cycles were conducted (10 mM tris-HCl buffer, pH 8.0) followed by incubation (45 °C, 24 h). Samples were treated, for 24 h, with 0.1% sodium dodecyl sulfate solution and washed twice with PBS. Decellularized cartilage samples were stored at −80 °C until use.

### 2.4. 3D Culture Models

**Figure 1:**
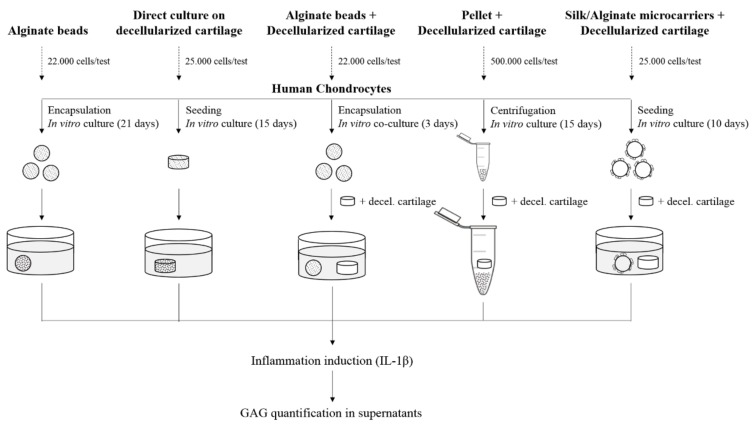
Schematic representation of study’s experimental design. Five 3D models were proposed considering the type of scaffolds, the number of cells/test, and the in vitro culture time as process parameters.

Five 3D models were considered: alginate beads (beads), decellularized cartilage (matrix), alginate beads and decellularized cartilage co-cultured (beads/matrix), pellet and decellularized cartilage co-cultured (pellet/matrix), and silk/alginate microcarriers and decellularized cartilage (microcarriers/matrix) (Figure 1). For beads model we considered ACs and NCs while, for other models, we used only NCs. Samples were treated with interleukin 1β (IL-1β, 10 ng/mL, 48 h), to induce an inflammation-like status. In order to eliminate the variability of the IL-1β activity, we used the same cytokine batch for all experiments. Supernatants were analyzed quantifying GAG content.

#### 2.4.1. Alginate Beads

Encapsulation of ACs (6 cell lines from six donors) and NCs (six cell lines from six donors) was performed as reported by [12,13], with some modifications. Briefly, for each batch, cells (10^6^ cells/mL) were suspended in a sodium alginate solution (1% *w*/*v*) that was then added drop-wise, with a needle (21 G), into a 0.9% NaCl solution containing CaCl_2_ 50 mM. Constructs were collected by filtration through a nylon mesh (Falcon^TM^, Corning, New York, NY, USA) with 100 µm pores, rinsed with NaCl solution, and re-suspended in culture medium. Each alginate bead contained 22,155 ± 6119 cells (*n* = 25).

For beads model, constructs were cultured for at least 21 days before IL-1β treatment. Beads were cryopreserved in freezing media containing 10% of DMSO. Constructs were incubated for 3 h in freezing media at 4 °C in order to allow penetration of DMSO.

#### 2.4.2. Direct Culture on Decellularized Cartilage

NCs (three cell lines from three donors) were suspended in culture medium: 50 µL of cell suspension (25,000 cells) were added to each decellularized matrix segment (2 mm). After 2 h of incubation, culture medium was added and constructs were cultured for 15 days to obtain the scaffold colonization before the inflammation induction. Media were replaced every 2–3 days.

#### 2.4.3. Alginate Beads and Decellularized Cartilage

Alginate beads were produced as previously reported; we overall produced six bead batches considering six nasal septal-derived cell lines (NCs) obtained from six different donors. For this 3D model, constructs were produced, cultured for three days in chondrogenic medium and finally cryopreserved. Before the use, beads were subjected to a rapid thawing and co-cultured for three days with decellularized cartilage (2 mm) prior the inflammation induction.

#### 2.4.4. Pellet and Decellularized Cartilage

Pellet culture were performed as described by [14], with some modifications. Aliquots of NCs (0.5 × 10^6^ cells) were centrifuged (300× *g*, 5 min) and incubated for 24 h. After this period, each tube was filled with 1 mL of medium. Medium was then replaced every two days; after 15 days of culture, in each tube, was added decellularized cartilage segment and then samples were treated with pro-inflammatory cytokine IL-1β. This condition was tested in triplicate considering three NC cell lines from three donors.

#### 2.4.5. Silk/Alginate Microcarriers and Decellularized Cartilage

Microcarriers were prepared using a bead generator (VAR V1, Nisco Engineering AG, Züric, Switzerland), as reported by [15,16]: 1% *w*/*v* sodium alginate solution was added dropwise by a syringe pump (Nisco Engineering AG, Züric, Switzerland), using a micro-nozzle (internal diameter 0.17 mm) and applying a differential charge of 7 kV, into an aqueous solution of CaCl_2_ (100 mM). Alginate microcarriers were collected and washed with distilled water. The obtained microcarriers were shaken into a silk fibroin solution (1.5% *w*/*v*) under magnetic stirring for 5 min and, subsequently, collected by filtration and immersed in 96% *v*/*v* ethanol (Carlo Erba, Cornaredo, Italy) to induce silk conformational transition. This procedure was repeated for three times. Silk/alginate microcarriers were lyophilized; before the use microcarriers were conditioned with culture medium overnight. NCs were added to conditioned carriers (0.85 mg) in a tube, at a density of 25,000 cells/sample. Tube was stirred on an oscillating shaker (Rotamax 120, Heidolph Inst. GmbH and Co, Schwabach, Germany) for 2 h at 70 rpm to allow cell seeding. After the seeding step, fresh medium was added and tube was incubated for 10 days, replacing culture medium every three days. Microcarriers were subjected to cryopreservation until use. Before use, microcarriers were thawed and cultured for three days with decellularized matrix segments prior to inflammation induction. Three batches of microcarriers were produced considering three cell lines (NCs) from three donors.

### 2.5. In Vitro Assays

#### 2.5.1. Glycosaminoglycans Quantification

Glycosaminoglycans (GAG) quantification has been performed using the 1,9-dimethylmethylene blue (DMB) dye method as reported by [17]. For beads model, cartilage matrix produced by chondrocytes was digested overnight at 60 °C, using papain solution (0.6 U/mL). Then, 40 µL of digested sample solution and 250 µL of DMB solution were analyzed spectrophotometrically. For other 3D models GAG were quantified in supernatants because inflammatory stimuli produced degeneration of proteoglycans that released GAG in culture media. Chondroitin-6-sulfate was considered as standard: a standard curve (*r*^2^ = 0.99) was obtained analyzing eight sequential dilutions (0–50 µg/mL). The absorbance was detected at 595 nm using a microplate reader. Each condition was tested in triplicate.

#### 2.5.2. Cell Metabolic Activity Evaluation (MTT Assay)

MTT assay was performed for each 3D model, before and after IL-1β treatment (10 ng/mL, 48 h), to evaluate the effect of inflammatory-like stimulation on NCs metabolic activity. For each 3D model (beads, direct culture on decellularized cartilage, beads + decellularized cartilage, pellet + decellularized cartilage and microcarrier + decellularized cartilage), the same three cell lines (NCs) were considered to include the donor variability in the experimental design. Moreover, MTT assay was performed before and after thawing, only for samples cryopreserved (beads and microcarrier, both with decellularized matrix).

Each scaffold was incubated with 100 µL of 3-(4,5-dimethylthiazol-2-yl)-2,5-diphenyltetrazolium bromide (MTT) solution (0.5 mg/mL). After 3 h, MTT solution was removed and the sample was mixed with DMSO to assure the solubilization of formazan crystals. Optical density (OD) of DMSO solution was measured on FLUOstar Omega (BMG Labtech, Ortenberg, Germany) at 570 nm and 670 nm (reference wavelength). Relative cell metabolic activity (%) was calculated as follows: 100×(ODs/ODc), where ODs represents the mean value of OD of tested sample and ODc is the mean value of OD of untreated cells (control). Each condition was tested in triplicate.

#### 2.5.3. Histological Investigation

Bead constructs, cultured for 35 days and cryopreserved, were placed in specific plastic biocassettes (Thermo Scientific, Waltham, MA, USA), routinely processed and then embedded in paraffin. Four micrometer-thick sections were cut and stained with haematoxylin and eosin (H and E). To evaluate functionality of chondrocytes, further sections were immunostained with anti-collagen antibodies (Abcam, Cambridge, UK). All reactions were developed with EnVision + System-HRP (DAB) (DAKO, Stockport, UK).

### 2.6. Statistical Analysis

Cellular doubling data were processed by an Analysis of Variance (ANOVA), considering cell source (articular or nasal), cell lines and culture passage as independent variables. Replication rate and population doubling were processed by ANOVA, considering cell source (articular or nasal) and cell line as independent variables and culture time as covariate variable. For each considered 3D model, GAG production results (µg GAG/mL) were analyzed by two-way ANOVA to assess the effect of culture time and IL-1β treatment, considered as independent variables. The differences between groups were analyzed with post hoc LSD’s test for multiple comparisons. Data are expressed as mean ± standard deviation. Statistical significance was fixed at *p* < 0.05. MTT assay results were analyzed by ANOVA, considering IL-1β treatment, 3D model and cell lines as independent variables, and relative cell metabolic activity as response variable. Data are reported as mean values ± standard deviation. Statistical significance was fixed at *p* < 0.05.

## 3. Results

**Figure 2:**
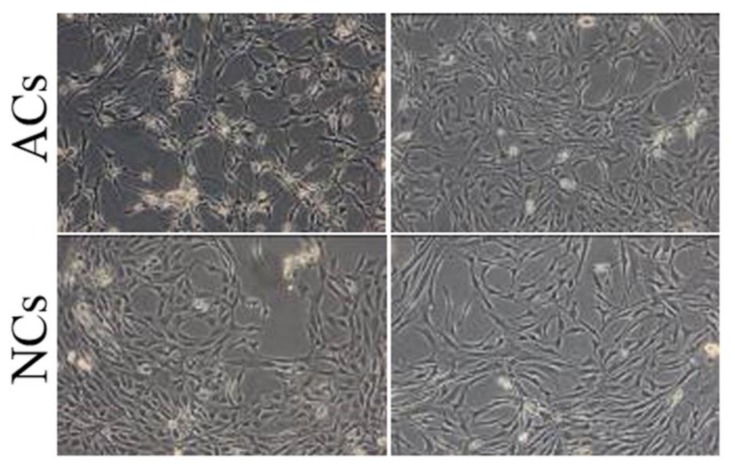
Optical microscope images of two different cell lines of articular (ACs) and nasal septal (NCs) chondrocytes cultured in monolayer condition until sub-confluence for eight and 14 days, respectively (25× magnification). Cells appeared as spindle-shaped without morphological differences.

In cartilage tissue engineering field, a critical factor is the achievement of an adequate number of functional chondrocytes for the complete colonization of scaffolds; in this context, the main limit is represented by the low number of cells obtained from donor tissues and by their easy loss of phenotype that causes the loss of functionality. We considered articular (ACs) and nasal septum (NCs) chondrocytes to evaluate the optimal cell source for the production of engineered cartilage tissue in a short time. There are no differences (*p* > 0.05) in terms of cellularity between articular (3.60 × 10^6^ cells/g of tissue) and nasal septal cartilage (3.97 × 10^6^ cells/g of tissue). Both cell lines, cultured in monolayer condition, appeared as spindle shaped without morphological differences (Figure 2); however, at the same culture condition, ACs and NCs reached 70% of confluency in 14 and eight days, respectively.

Cellular doubling results, obtained considering cell lines from different donors, and cell sources (ACs and NCs) as independent variables, indicated that NCs exhibited higher values of CD (3.4 ± 0.2) than ACs (1.9 ± 0.2) (*p* < 0.0001). Moreover, statistical analysis demonstrated that replication rate and population doubling time, calculated during exponential growth, were independent from the cell donor, but were significantly influenced by the cell source (higher values of RR and PD for ACs with respect to NCs, *p* = 0.0007). In particular, RR results were 28.23 ± 12.869 and 6.58 ± 7.317 for ACs and NCs, respectively; the same trend was obtained for PD results (8.528 ± 3.888 and 1.988 ± 2.210 for ACs and NCs, respectively).

### 3.1. Alginate Beads

**Figure 3:**
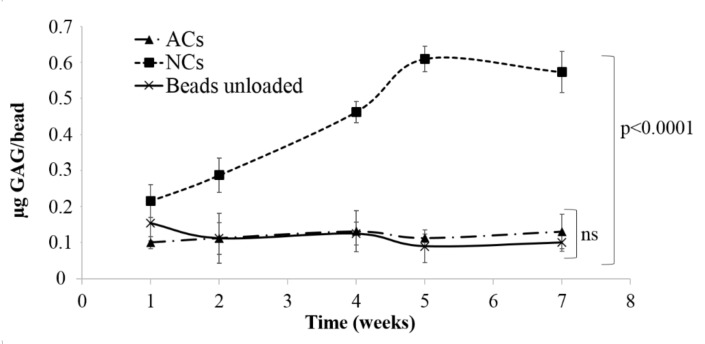
Mean values and standard deviation of µg GAG/bead, produced by encapsulated articular (ACs) and nasal (NCs) chondrocytes after 1, 2, 4, 5, and 7 weeks of culture. At each considered time, three beads were analyzed to determine the GAG production; overall, six cell lines were considered for ACs and six cell lines were considered for NCs. Unloaded beads were considered as the negative control (experimental sampe size = 90).

ACs and NCs were encapsulated in alginate beads and cultured for seven weeks to allow cartilage matrix production. At basal state (no inflammation) GAG analysis were conducted on constructs to monitor the production of extracellular matrix (ECM). Results are expressed as the amount of GAG detected for beads, considering unloaded beads as the control. Statistical analysis demonstrated a significant effect of cell donor on GAG production (*p* < 0.05), both for ACs and NCs. Results indicated that NCs were able to produce quantifiable cartilage matrix already after two weeks of culture (Figure 3) and GAG content increased during that time (0.43 ± 0.02 µg GAG/bead). At the same time, no significant differences were observed between encapsulated ACs (0.12 ± 0.05 µg GAG/bead) and unloaded beads (0.10 ± 0.02 µg GAG/bead) during all considered period (Figure 3).

For this reason, ACs were not suitable for this model and further tests were conducted on NCs-based constructs after 21 days of culture, the time necessary to produce a quantifiable cartilage matrix in vitro. NCs-based constructs were treated with pro-inflammatory cytokine IL-1β and GAGs released in culture supernatants (µg of GAG/mL) were quantified. Results indicated that IL-1β treatment did not produce an increase of GAGs (*p* > 0.05) in supernatants (0.01 ± 0.12 and 0.03 ± 0.12 µg GAG/mL in untreated and inflamed cells, respectively). These results evidenced that encapsulated chondrocytes produced and released, after inflammation induction, low amounts of GAGs; for this reason, this 3D model was not suitable for the development of in vitro cartilage degeneration.

### 3.2. Direct Culture on Decellularized Cartilage

In order to overcome the problem related to the low amount of cartilage matrix produced in vitro by the encapsulated chondrocytes, we used decellularized human cartilage as the 3D scaffold for chondrocyte cultures and as aggrecanase substrate. After decellularization process, cells were dead and only cell fragments were visible (data not shown). Chondrocytes were seeded on decellularized matrix, cultured for 15 days (time necessary to obtain the complete scaffold colonization) and then were subjected to the treatment with pro-inflammatory cytokines. Supernatants of samples treated with IL-1β showed a higher amount of glycosaminoglycans (29.37 ± 16.13 µg GAG/mL) than samples in basal medium (2.88 ± 1.29 µg GAG/mL) (*p* < 0.05).

### 3.3. Alginate Beads and Decellularized Cartilage

**Figure 4:**
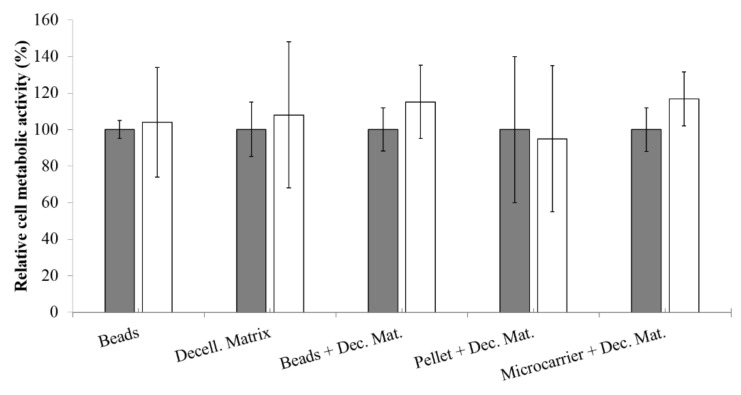
Relative cell metabolic activity (percentage) of chondrocytes for each 3D model before (grey bars) and after (white bars) stimulus with pro-inflammatory cytokine IL-1β (48 h). Mean values obtained from three cell lines ± standard deviations. Each condition was tested in triplicate (experimental sample size = 90).

NCs were encapsulated and cultured for three days. Each bead was put into a well with a decellularized matrix segment and treated with IL-1β to induce aggrecanase release by the chondrocytes and the subsequent degradation of cartilage matrix. Results showed that GAGs released in supernatants by treated cells were significantly higher (*p* = 0.041) than untreated cells (7.252 ± 0.46 and 1.55 ± 0.33 µg GAG/mL, respectively). The cell viability of encapsulated NCs was assessed with MTT assay in order to evaluate the effect of pro-inflammatory cytokine treatment (Figure 4); data demonstrated that chondrocytes stimulated for 24 h with pro-inflammatory cytokine presented higher metabolic activity (*p* < 0.05) with respect to untreated cells (data not shown). On the other side, after 48 h of culture, no differences (*p* > 0.05) were found between the two treatment groups (Figure 4, treated and untreated with IL-1β).

### 3.4. Pellet and Decellularized Cartilage

Pellet of NCs, cultured for 15 days, were inflamed in presence of decellularized cartilage matrix. IL-1β treatment promoted the cartilage matrix degradation (14.73 ± 0.87 µg/mL) with respect to untreated cells (9.79 ± 0.87 µg/mL) (*p* = 0.002).

### 3.5. Silk/Alginate Microcarriers and Decellularized Cartilage

Microcarriers were loaded with NCs and then cultured for 10 days to allow cell adhesion and growth. After treatment with IL-1β, results showed that GAGs released were significantly higher (*p* = 0.0003) for inflamed group with respect to control group (9.93 ± 1.06 and 3.35 ± 1.06 µg GAG/mL, respectively).

**Figure 5:**
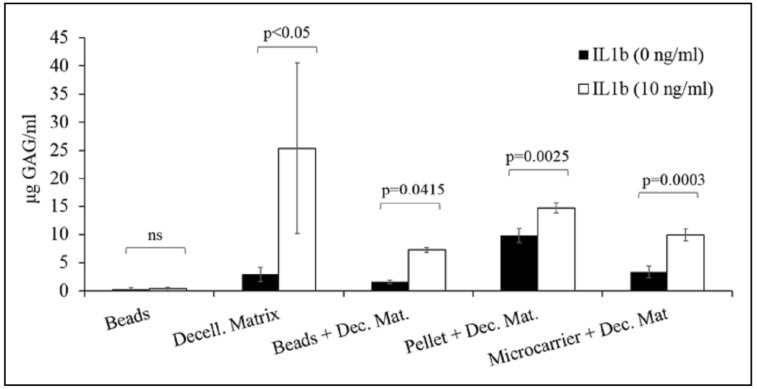
Mean values and standard deviation of µg GAG/mL released by NCs before and after the treatment with IL-1β, for each 3D model.

Statistic showed that all 3D culture models, except beads model, were suitable for the development of in vitro cartilage degradation, because the treatment with pro-inflammatory cytokine IL-1β induced the GAGs release in supernatants by nasal chondrocytes (Figure 5).

Considering decellularized cartilage as the scaffold for nasal chondrocyte growth, after IL-1β treatment, we detected the higher level of released GAGs; despite this, the 3D model showed different limits, including high variability in GAGs release and high number of cells required to obtain complete scaffold colonization. On the other side, the association of decellularized matrix, as aggrecanase substrate, and beads, pellet or microcarriers, used as scaffold for chondrocytes growth, allowed to obtain a significant increase of GAG release after treatment with pro-inflammatory cytokine IL-1β.

### 3.6. Relative Cell Metabolic Activity

Metabolic activity of NCs was determined with an MTT assay, as a function of pro-inflammatory cytokine treatment, for each considered 3D model (Figure 4). Statistical analysis showed that both variables (3D model and treatment) do not significantly influence NCs metabolic activity. However, after treatment with IL-1β, beads, decellularized matrix, and pellet + decellularized matrix models, presented an increase of data dispersion, indicating that these culture conditions lead to different metabolic activity depending on the sample statistical unit. On the other hand, for beads + decellularized matrix and microcarrier + decellularized matrix models, the treatment with IL-1β leads to lower data dispersion and to an increased metabolic activity (although not significant, *p* > 0.05). These results demonstrated that our models allowed us to preserve high cell metabolic activity during all experiments (both before and after the treatment with pro-inflammatory cytokine).

**Figure 6:**
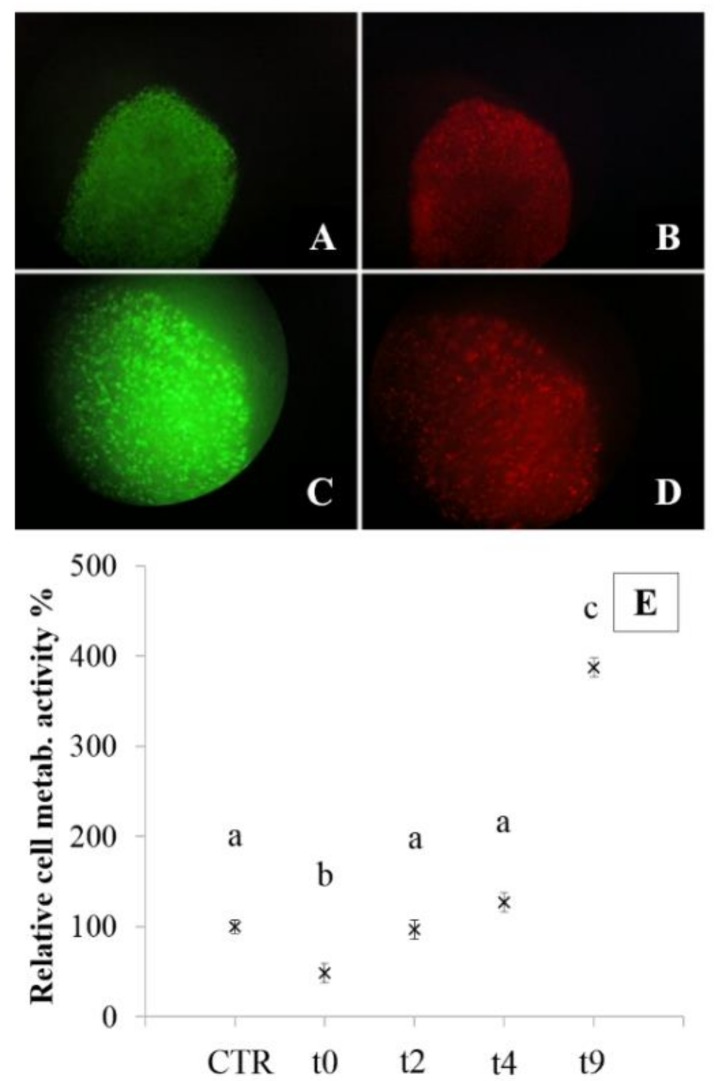
Live (green) and dead (red) staining of encapsulated NCs (beads model) after cryopreservation. Magnification at 5× (**A**,**B**) and 10× (**C**,**D**). Relative cell metabolic activity % (**E**), expressed as mean values ± standard deviation, of encapsulated NCs before cryopreservation (control) and after the thawing process (t0-2-4-9 days). Different letters indicate significant differences (*p* < 0.05). Overall we considered three bead batches produced from three nasal chondrocyte lines, and, for each considered time, three beads were analyzed (experimental sample size *n* = 45).

**Figure 7:**
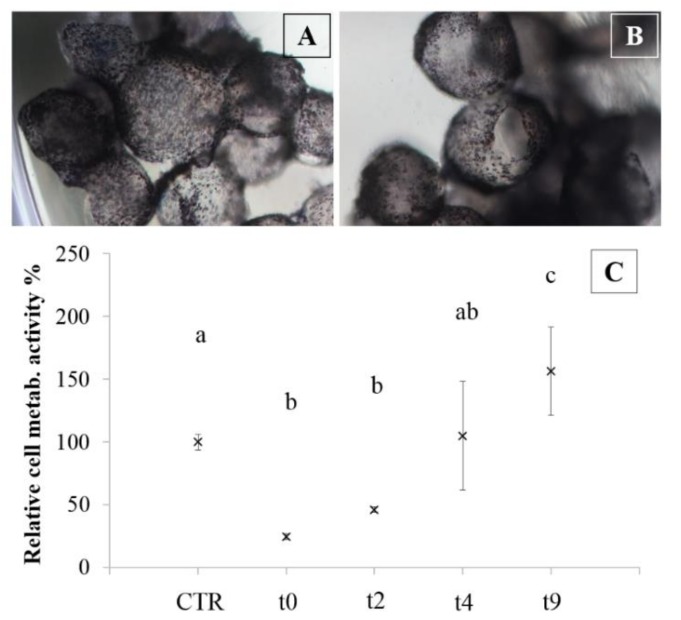
Formazan staining for viable cells on silk/alginate microcarriers, before cryopreservation (**A**) and seven days after thawing (**B**). Magnification of 5×. Relative cell metabolic activity % (**C**), expressed as mean values ± standard deviation of encapsulated chondrocytes before cryopreservation (control) and after thawing process (t0-2-4-9 days). Different letters indicate significant differences (*p* < 0.05). Overall we considered three bead batches, produced from three nasal chondrocyte lines and, for each considered time, three beads were analyzed (experimental sample size *n* = 45).

Metabolic activity was also evaluated for nasal chondrocytes, loaded in alginate beads and on alginate-based microcarriers, after cryopreservation (Figure 6 and Figure 7). Cryopreservation of alginate constructs is the major feature of these models, because it would make possible to prepare, characterize and store constructs until use.

In particular, the bead model is the most useful for this purpose because encapsulation preserves different cell lines from cryopreservation damage. The MTT test, performed before cryopreservation (control) and after thawing (0, 2, 4, and 9 days of culture) on encapsulated NCs, showed that post-thawing, two days of culture are required to resume cell metabolism at basal state (Figure 6E). Within four days from thawing, cell metabolism was significantly higher with respect to control cells (*p* < 0.05). These results were also confirmed by live-dead assay, that confirmed 90% of cell viability after two days of culture (Figure 6A,C live cells, Figure 6B,D dead cells).

The cryopreservation process was also performed on microcarriers; Figure 7 shows images of microcarriers treated with MTT solution before (Figure 7A) and after the freezing-thawing protocol (Figure 7B). Formazan staining (violet) marked viable cells and confirmed that there were no differences between cells before the cryopreservation and after seven days post-thawing. Furthermore, in Figure 7C, an increase of chondrocyte metabolic activity was reported in the period between two and nine days of culture after thawing.

These results confirmed that alginate-based scaffolds (both beads and microcarriers) could be considered as good vehicles for NCs because they are able to protect the cells from the damage induced during the freezing process. Furthermore, beads and microcarriers scaffolds, combined with decellularized matrix, allowed us to obtain models that response to the inflammatory stimulus and, on the other side, significantly reduce the number of cells and the culture time with respect to the direct culture on decellularized matrix and pellet (Figure 1).

### 3.7. Histological Investigation

**Figure 8:**
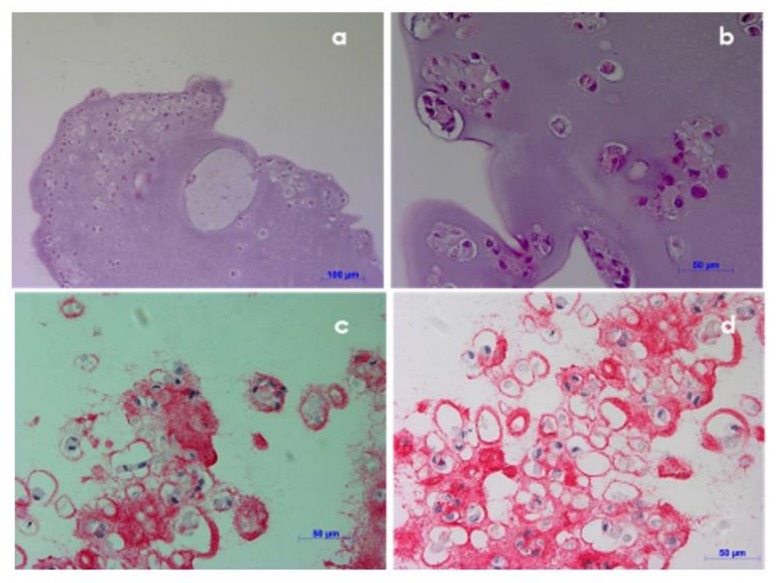
Histological evaluation of encapsulated NCs (beads model) after 35 days of culture post cryopreservation: Hematoxylin-eosin staining (**a**,**b**) and immunostaining for type II collagen (the phenotypic marker for chondrocytes) (**c**,**d**).

Histological investigation was performed on NCs encapsulated in alginate beads cultured for 35 days, cryopreserved and then thawed, to evaluate cell morphology and functionality. Hematoxylin-eosin staining showed that cells were spherical, with shaped-central nucleus and basophilic cytoplasm, similar to the natural cartilage morphology (Figure 8A,B). Moreover, cryopreserved constructs presented type II collagen that is a phenotypic staining of cartilage matrix, confirming the cell functionality after cryopreservation (Figure 8C,D).

## 4. Discussion

In this study, five in vitro 3D models were proposed to perform a reproducible, robust, well-defined, and cost-affordable model of osteoarthritis, one of the most common pathologies that affects cartilage tissue. This research was focused on the development of engineered cartilage, in order to test a large number of 3D models, using GAG quantification as the principal response variable. This approach allowed us to reduce the time and cost of analyses for the first screening of 3D models. The proposed approach represents a cheap, easy, and fast spectrophotometric analysis that could be exploited in early stages of drug discovery for the screening of new potential Active Pharmaceutical Ingredient (API).

Human articular and nasal chondrocytes were selected as cell sources, and IL-1β was used to induce cartilage tissue degeneration. The development of a cartilage degeneration model that mimics in vivo pathological conditions could be used in drug screening processes, reducing time and costs related to the use of animal models. Our results indicated that expansion rate of NCs was higher with respect to ACs, in accordance to [18]. Furthermore, considering alginate beads model, we demonstrated that GAGs production was significantly higher in NC cultures compared to ACs and we conducted the cell culture until obtaining a quantifiable cartilage matrix (seven weeks). Different authors confirmed that NCs show superior and more reproducible in vitro chondrogenic potential [7,18,19,20]; this is possibly due to the pathological condition of articular tissues that are commonly harvested in patients with osteoarthritis or after sport trauma (during joint replacement surgery) [21,22]. These patients were typically treated with cortisone and other anti-inflammatory drugs, which are able to partially suppress the inflammatory cascade but, at the same time, could have an inhibition effect on articular chondrocyte proliferation. As previously reported by other researchers, nasal cartilage can be considered as a chondrocyte source because the tissue is made by hyaline cartilage containing differentiated chondrocytes able to express the typical collagen types of articular cartilage [23,24]. Furthermore, Kafienah and colleagues [18] demonstrated that both articular and nasal septal chondrocytes were able to yield a matrix that lacks the organization of natural articular cartilage. This result suggested that the complex organization of articular cartilage is not correlated to the property of selected cells (articular or nasal) but may be rather ascribed to the tissue remodeling after in vivo physiological loading conditions. For these reasons, we selected septal nasal tissues as the chondrocyte source in order to reduce cell culture time, with consequent reduction of phenotype loss risk correlated to monolayer culture conditions.

The use of alginate beads as 3D scaffold for engineered cartilage production has been already proposed [7,25]: cells are dispersed in matrix to avoid the formation of necrotic centers (like the pellet culture), and allowed to grow alone or in small clumps. One of advantages of this culture technique is that, with a chelating solution, it is possible to dissolve the alginate matrix to recover cells and quantify them; at the same time alginate bead cultures for long periods did not influence the maintenance of the phenotypic profile [26,27]. Despite this, our results demonstrated that encapsulated chondrocytes were able to produce a small amount of ECM (GAGs) only after high culture times (at least three weeks). In order to reduce the time necessary to obtain the engineered tissue, we tested decellularized nasal septal cartilage, which has been proposed as a promising biomaterial for cartilage regeneration [28]. In our research the direct culture of chondrocytes on the decellularized matrix made it possible to produce a degeneration model of cartilage within 15 days; the low porosity of the decellularized matrix was the main cause of limited cell infiltration and of slow cell colonization [28]. For these reasons, it is necessary to provide the in vitro culture two weeks before the induction of inflammation.

Based on this evidence, we hypothesized that the decellularized cartilage matrix could be used only as a substrate of aggrecanase action. Here we proposed, for the first time, the combined use of the decellularized matrix with 3D culture methods (pellet, alginate beads, and silk/alginate microcarriers). The pellet is the common method used for 3D culture of chondrocytes, which is obtained by cell centrifugation to form a micromass [29,30]. In accordance with other researchers [31,32] we demonstrated that pellet culture maintains chondrocyte viability and their ability to secrete aggrecanase after treatment with pro-inflammatory cytokine IL-1β; despite that, this culture method presents some limitations, mainly correlated to high number of cells per test (500,000 cells), and high culture time (15 days) required. In addition, Randau and colleagues [33] demonstrated that the pellet culture induces the cell necrosis after 15 days of culture; this evidence could explain the results obtained in our study. In fact, we quantified high GAG levels released by cells also in basal condition (without pro-inflammatory stimulus). Therefore, we supposed that chondrocytes cultured in pellets were stressed and, thus, released pro-inflammatory bioactive compounds able to degrade the extracellular matrix. Chondrocyte culture in alginate bead scaffolds is a method used to obtain in vitro cartilage-like matrix; mechanical and structural characteristics of alginate provide a micro-environment similar to native tissue [34]. Results showed that it is possible to use bead model in combination with decellularized cartilage to induce inflammatory response of cells, although this effect is not particularly visible, like other considered models. This could be due to the size-exclusion effect of alginate beads that partially retain aggrecanase in alginate matrix. Tanaka and collaborators [35] demonstrated that alginate membrane is able to retain molecules up to 68 kDa; aggrecanase has a molecular weight of about 70 kDa and it is possible that alginate beads cannot release these enzymes, preventing the matrix degradation. To overcome these limitations, we proposed silk/alginate microcarriers as scaffold: chondrocytes grow on the particle surface and are not entrapped into alginate matrix. This kind of structure allows 3D growing that mechanically stimulates chondrocytes to retain specific phenotype [32]. 3D culture on microcarrier surface was allowed by silk treatment, which anchors cells and allows culture without de-differentiation. Moreover, it is possible to avoid the sieve effect of alginate matrix [36]. Different authors demonstrated that beads are able to protect different kinds of cells against freezing damages [12,37,38]. Our results confirm that it is possible to cryopreserve chondrocytes in alginate beads, because after thawing they progressively resume the metabolic activity and maintain phenotypic markers, such as collagen type II. Similar results were obtained from Almqvist [39] that preserved encapsulated chondrocytes at −196 °C for 24 h without a significant decrease in aggrecan synthesis rate. Likewise, chondrocytes grown on microcarriers were cryopreserved and results, obtained after thawing, demonstrated that cells were viable and neo-tissue maintained typical structure, as observed on microscope. Furthermore, microcarriers and alginate beads could be cultured in a fluid perfusion system in order to enhance cell growth, productivity, and to maximize the efficiency of scaffold-based culture. Based on this, we will consider this culture method for the subsequent process scale-up phase. Moreover, for subsequent final model validation step in a European Reference Point, according to EU 2010/63/EU and IT 2014/26, the aggrecanase quantification will be assessed by RT-PCR, and the efficacy of anti-inflammatory drugs on the selected 3D model will be tested.

## 5. Conclusions

Here we proposed, for the first time, the combined use of decellularized cartilage, as an aggrecanase substrate, with pellet, alginate beads, or silk/alginate microcarriers, as scaffolds for chondrocyte in vitro cultures. This approach allowed us to obtain an osteoarthritis model that responds to IL-1β-induced inflammatory stimulus. Despite this, the pellet model requires a high number of cells and high culture time with respect to alginate-based scaffolds and, in addition, bead and microcarrier models can be stored by cryopreservation since chondrocyte functionality and metabolic activity are maintained after thawing. For these reasons, we can conclude that alginate and silk/alginate-based scaffolds are storable and “ready-to-use” products for screening of new drugs for osteoarthritis in early stages of drug discovery.
